# A cluster randomized clinical trial to evaluate the effectiveness of the Implementation of Infant Pain Practice Change (ImPaC) Resource to improve pain practices in hospitalized infants: a study protocol

**DOI:** 10.1186/s13063-019-3782-9

**Published:** 2020-01-06

**Authors:** Mariana Bueno, Bonnie Stevens, Melanie A. Barwick, Shirine Riahi, Shelly-Anne Li, Alexa Lanese, Andrew R. Willan, Anne Synnes, Carole A. Estabrooks, Christine T. Chambers, Denise Harrison, Janet Yamada, Jennifer Stinson, Marsha Campbell-Yeo, Melanie Noel, Sharyn Gibbins, Sylvie LeMay, Wanrudee Isaranuwatchai

**Affiliations:** 1 Child Health and Evaluative Sciences, The Hospital for Sick Children, Peter Gilgan Centre for Research and Learning (PGCRL), 686 Bay Street, 6th floor, Toronto, M5G 0A4 Canada; 2 Lawrence S. Bloomberg Faculty of Nursing & Faculties of Medicine and Dentistry, University of Toronto, 155 College Street, M5T 1P8 Toronto, Canada; 3 Department of Psychiatry, Faculty of Medicine, University of Toronto, 250 College Street, M5T 1R8 Toronto, Canada; 4 Dalla Lana School of Public Health, University of Toronto, 155 College Street, M5T 3M7 Toronto, Canada; 5 University of British Columbia, Pediatrics, Rm. 1N18, 4480 Oak Street, Vancouver, British Columbia V6H 3V4 Canada; 6 University of Alberta, Edmonton Health Clinic Academy, Rm 5-006 11405 87 Avenue NW, T6G 1C9 Edmonton, Alberta Canada; 7 Departments of Pediatrics and Psychology & Neuroscience, Dalhousie University and Centre for Pediatric Pain Research, IWK Health Centre, P.O. Box 9700 5850-5980 University Ave, Halifax, Nova Scotia B3K 6R8 Canada; 8 School of Nursing, Faculty of Health Sciences, University of Ottawa, and Children’s Hospital of Eastern Ontario, 401 Smyth Road, Ottawa, K1H 8L1 Canada; 9 Ryerson University, Daphne Cockwell School of Nursing, 350 Victoria Street, Toronto, Ontario M5B 2K3 Canada; 10 School of Nursing, Faculty of Health, Departments of Pediatrics and Psychology & Neuroscience, Dalhousie University and Centre for Pediatric Pain Research, IWK Health Centre, 5869 University Ave, Halifax, B3H 4R2 Canada; 11 Department of Psychology, University of Calgary, Psychology, Rm. 260, Administration Building, 539 Campus Place NW, T2N 4V8 Calgary, Canada; 12 Alberta Children’s Hospital Research Institute, Hotchkiss Brain Institute, Owekro Centre, Calgary, Alberta Canada; 13 Trillium Health Partners, Professional Practice, 2200 Eglinton Ave W, Mississauga, Ontario L5M 2N1 Canada; 14 Université de Montréal, Faculty of Nursing and CHU Sainte-Justine’s Research Centre, 3175 Chemin de la Côte-Sainte-Catherine, Montreal, Quebec H3T 1C5 Canada; 15 Institute for Health Policy, Management and Evaluation, University of Toronto, 155 College Street, M5T 3M7 Toronto, Canada; 16 St. Michael’s Hospital, 30 Bond Street, Toronto, Ontario M5B 1W8 Canada

**Keywords:** Pain, Procedural, Infants, Assessment, Management, Implementation, Context

## Abstract

**Background:**

Hospitalized infants undergo multiple painful procedures daily. Despite the significant evidence, procedural pain assessment and management continues to be suboptimal. Repetitive and untreated pain at this vital developmental juncture is associated with negative behavioral and neurodevelopmental consequences. To address this knowledge to practice gap, we developed the web-based Implementation of Infant Pain Practice Change (ImPaC) Resource to guide change in healthcare professionals’ pain practice behaviors. This protocol describes the evaluation of the intervention effectiveness and implementation of the Resource and how organizational context influences outcomes.

**Methods:**

An effectiveness-implementation hybrid type 1 design, blending a cluster randomized clinical trial and a mixed-methods implementation study will be used. Eighteen Neonatal Intensive Care Units (NICUs) across Canada will be randomized to intervention (INT) or standard practice (SP) groups. NICUs in the INT group will receive the Resource for six months; those in the SP group will continue with practice as usual and will be offered the Resource after a six-month waiting period. Data analysts will be blinded to group allocation. To address the intervention effectiveness, the INT and SP groups will be compared on clinical outcomes including the proportion of infants who have procedural pain assessed and managed, and the frequency and nature of painful procedures. Data will be collected at baseline (before randomization) and at completion of the intervention (six months). Implementation outcomes (feasibility, fidelity, implementation cost, and reach) will be measured at completion of the intervention. Sustainability will be assessed at six and 12 months following the intervention. Organizational context will be assessed to examine its influence on intervention and implementation outcomes.

**Discussion:**

This mixed-methods study aims to determine the effectiveness and the implementation of a multifaceted online strategy for changing healthcare professionals’ pain practices for hospitalized infants. Implementation strategies that are easily and effectively implemented are important for sustained change. The results will inform healthcare professionals and decision-makers on how to address the challenges of implementing the Resource within various organizational contexts.

**Trial registration:**

ClinicalTrials.gov, NCT03825822. Registered 31 January 2019.

## Contributions to the literature

- Minimizing the research to practice gap in neonatal pain is imperative and timely. To our knowledge, the ImPaC Resource is the first online, multifaceted implementation tool for self-administration to foster change and improvement of pain practices in infants.
- Using an effectiveness-implementation hybrid type 1 design will enable the evaluation of clinical effectiveness while gathering information on implementation outcomes and has the potential to speed and improve the translation of evidence-based practices into clinical care.
- Exploring the effectiveness of the Resource will inform about this approach to practice change towards better neonatal pain assessment and management and health outcomes.

## Background

Hospitalized infants undergo 7–17 painful procedures per day [1], with sick and preterm infants undergoing the most. Although abundant and high-quality evidence on reliable pain assessment measures [2] and effective and safe analgesic strategies [3–6] exist, neonates continue to experience procedural pain with suboptimal management in the Neonatal Intensive Care Unit (NICU) [1, 7, 8]. Repetitive and untreated pain from procedures is associated with early [9, 10] and later changes in various health outcomes [11–14]. The increasing complexity of care in the NICU and the potential burden of negative growth and development in the early years of life signal an imperative to translate high-quality evidence for minimizing procedural pain and its effects into practice and to improve outcomes.

To increase effective implementation of knowledge to practice, we developed the Implementation of Infant Pain Practice Change (ImPaC) Resource (Resource). The goal of this web-based multifaceted implementation strategy is to support change in the pain practice behaviors of healthcare professionals (HCP). The Resource uses a standardized seven-step approach that guides developing a change team, measuring readiness for change, assessing current pain practices, reviewing current evidence, developing an aim statement, providing implementation strategies and templates for utilization in the practice setting, and enabling audit and progress monitoring. The Resource builds on previous findings on the evaluation of a real-time multifaceted implementation intervention “Evidence-based Practice for Improving Quality” (EPIQ) involving 32 hospital units within eight Canadian pediatric hospitals [15, 16]. The EPIQ intervention incorporated high-quality evidence and quality improvement (QI) methods using interactive knowledge translation (implementation) strategies (e.g. reminders, education, educational outreach, and audit and feedback). The 16 units that received the EPIQ intervention demonstrated statistically significant improved pain practices and clinical outcomes for hospitalized children when compared to the 16 units that continued with standard practices [16]. Organizational context influenced clinical outcomes [15] but EPIQ was not always considered feasible or cost-effective [17] and results were only partially sustained over 12–36 months [15, 16]. To address these limitations, the Resource was designed as a user-friendly, universally available, self-administered web-based tool to support hospital-based change agents.

This protocol describes the development, implementation, and evaluation of the Resource. The study was informed by the Consolidated Framework for Implementation Research (CFIR) that details key constructs associated with implementation success [18] and Proctors’ Implementation Outcome Taxonomy [19]. The usability of the Resource was tested with end-users in non-clinical scenarios and clinical situations and was demonstrated to be feasible, acceptable, comprehensive, and trustworthy [20, 21].

The goal of this study is to evaluate the effectiveness and the implementation of the Resource and explore the association of organizational context with clinical and implementation outcomes.

## Methods/Design

### Primary objective

To determine intervention effectiveness of the ImPaC Resource.

### Specific primary objectives

To determine the intervention effectiveness of the ImPaC Resource on the:

1. nature and frequency of painful procedures;
2. probability that an infant has (i) pain assessed with a validated pain measure and (ii) a pain treatment strategy (pharmacologic, physical) implemented during a painful procedure.

### Secondary objective

To determine the implementation effectiveness of the ImPaC Resource.

### Specific secondary objectives

To describe the implementation effectiveness of the ImPaC Resource in terms of ease of use (feasibility), to complete as intended (fidelity), economically attractive (implementation cost), easily integrated into the NICU’s practice (reach), and sustainable (continued use with fidelity).

### Other objectives

To explore the influence of organizational context on clinical and implementation outcomes.

### Study design

The study will use an effectiveness-implementation hybrid type 1 design. This design tests the intervention effectiveness while gathering information on implementation issues [13]. Hybrid designs have the potential to move research forward at a pace that better fits changing eHealth technology while maintaining a thorough examination of intervention effectiveness [22, 23].

**Fig. 1:**
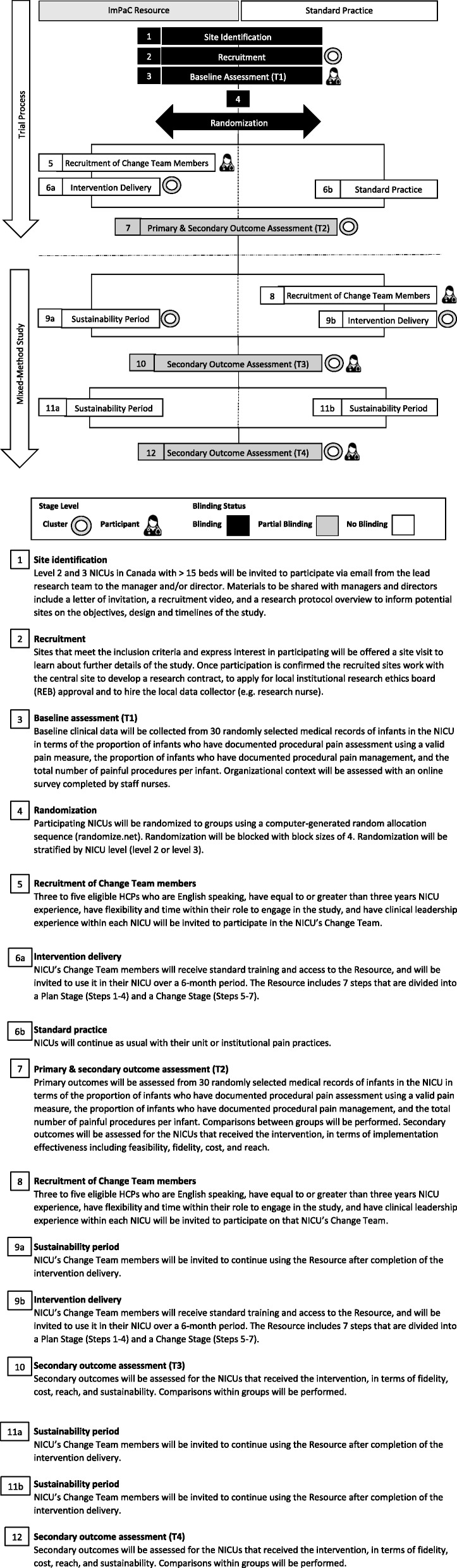
Timeline cluster *diagram* [35]

The intervention effectiveness will be assessed using a cluster parallel randomized clinical trial (RCT) where NICUs will be randomized to the intervention (INT) group or the standard practice (SP) group (Fig. 1). The SP group will be offered the Resource following completion of the intervention by the INT group.

Primary outcomes will be assessed between groups before and after the intervention and will be obtained from clinical medical records (i.e. chart review).

Secondary outcomes will be assessed within groups, in a mixed-methods descriptive study using qualitative (i.e. focus group) and quantitative (i.e. chart review, survey, metrics captured by the Resource website) data.

The influence of organizational context will be assessed using a quantitative data collection approach (e.g. chart review, survey, metrics captured by the Resource website).

**Fig. 2:**
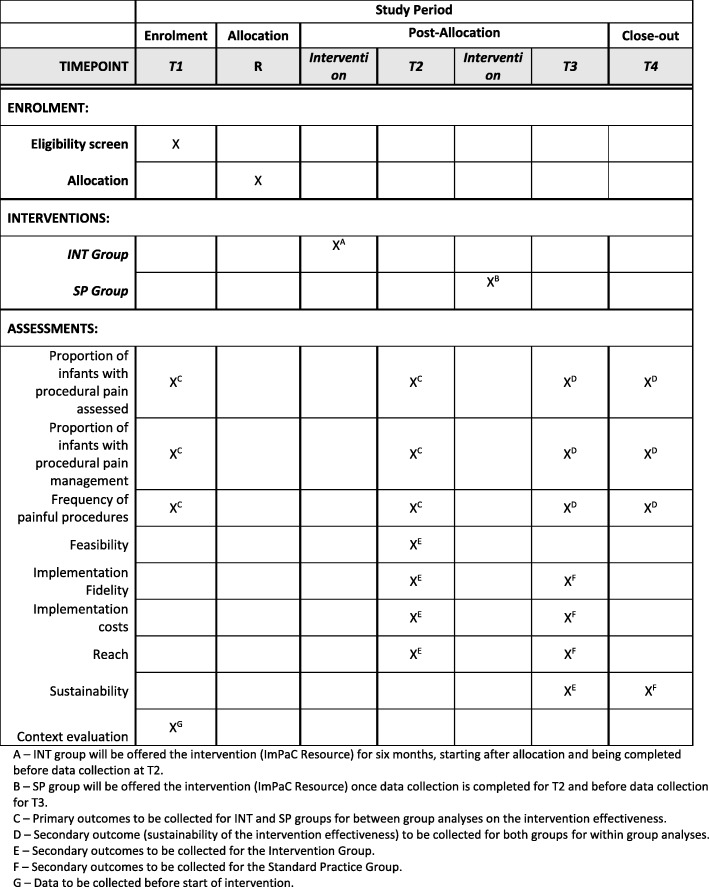
Schedule of enrolment, interventions, and assessment

Data collection timepoints (T) are included in Fig. 1. A schedule of enrolment, interventions, and assessment is included in Fig. 2. This study protocol follows the SPIRIT-C 2019 recommendations for intervention trials - Child Health Extension Checklist (Additional file 1).

### Setting

Level 2 or 3 NICUs in pediatric or general hospitals across Canada will be invited to participate. These units care for moderate-risk (Level 2) to high-risk (Level 3) neonates in need of respiratory support, from supplementary oxygen and positive airway pressure ventilation (Level 2) to highly invasive ventilation (Level 3) [24]. Units will be eligible if they: (1) have a minimum of 15 beds; and (2) agree to be engaged in the study for up to 24 months. Eighteen eligible units who agree to participate and obtain research ethics approval will be randomly assigned to either the INT or the SP groups.

### Participants

At each data collection point, hospital medical records will be reviewed for 30 infants who are hospitalized in the NICU for at least 24 uninterrupted hours. At each time point, the data collection from 30 infant medical records will be completed over a period of 1–4 weeks.

Staff in each participating NICU will be invited to participate in the Resource Change Team (who lead the implementation of the Resource) if they are a HCP, English speaking, have ≥ 3 years of NICU experience, have flexibility and time within their role to engage in the study, and have clinical leadership experience (e.g. in an advanced practice or clinical education role). The Change Team will be composed of 3–5 HCPs who will engage with the NICU nursing staff throughout the study.

NICU nursing staff members who are not part of the Change Team will be invited to complete the organizational context survey if they have ≥ 6 months of NICU experience and their full-time equivalent status is ≥ 0.5.

### Clinical (primary) outcomes

Clinical outcomes to determine the intervention effectiveness of the Resource will be retrieved from a standardized 24-h period from medical records of hospitalized infants at baseline (T1) and at six (T2) months after randomization for both groups (See Table 1), including:

1. The proportion of infants in the NICU who have documented procedural pain assessment using a valid pain measure (e.g. Premature Infant Pain Profile – Revised [25], Neonatal Infant Pain Scale [26], Behavioral Indicators of Infant Pain [27], among other valid measures each unit my use as part of their pain practices);
2. The proportion of infants in the NICU who have documented procedural pain management with evidence-based pharmacological or physical interventions documented;
3. Total number of documented painful procedures (e.g. heel lance, arterial puncture, eye examination) per infant in the NICU.

**Table 1:** Study primary, secondary, and other outcomes

Primary outcomes	Description	Measure/Metrics	Timepoints for data collection
Proportion of infants with procedural pain assessed	Total number of pain assessments performed using a valid pain measure and documented over a 24-h period (i.e. midnight to midnight)	Whether or not an infant’s procedural pain was assessed with a valid pain measure [7]	T1, T2 (for both groups)
Proportion of infants with procedural pain management	Total number of pain management interventions (pharmacologic and/or non-pharmacologic) implemented and documented over a 24-h period (i.e. midnight to midnight)	Whether or not an infant received pharmacologic (e.g. sucrose, opioids, acetaminophen) and/or non-pharmacologic (e.g. breastfeeding, skin-to-skin contact, non-nutritive sucking) interventions for procedural pain [7]	T1, T2 (for both groups)
Frequency of painful procedures	Total number of painful procedures documented in clinical charts over a 24-h period (i.e. midnight to midnight)	Absolute number of painful procedures per infant documented in a 24-h period [7]	T1, T2 (for both groups)
Secondary outcomes	Description	Measure/Metrics	Timepoints for data collection
Feasibility	The extent to which the Resource was successfully implemented in terms of ease of use and time [10, 15]	Focus group. A semi-structured interview guide was developed according to CFIR constructs on intervention characteristics [9] and has been pre-tested in a usability study [12]	T2 (for INT group)
Implementation fidelity	The degree to which the Resource is used by a team as prescribed or intended [10, 15]	Progression through the Resource, which includes completion of each of the seven steps, completeness of included information will be considered the metric for fidelity. These data will be captured from the backend of the website at completion of using the Resource	T2 and T3 (for INT and SP groups, respectively)
Implementation costs	The financial and time cost to implement the Resource [10]	Data on human resources (time spent on orientation session, navigation through the Resource, meeting among team members, implementation of the Resource either individually or in group); space (for meetings and education sessions, for example); equipment (e.g. laptop); materials (e.g. printing materials), and other related expenses. These data will be captured from the backend of the website.	T2 and T3 (for INT and SP groups, respectively)
Reach	The integration of a KT strategy within a service setting and its subsystems [15]	Proportion (%) of NICU staff who receive a KT strategy (selected from the Resource) divided by the total number of NICU staff expected to receive the service [15]. These data will be captured from the backend of the website	T2 and T3 (for INT and SP groups, respectively)
Sustainability	The extent to which the newly implemented Resource is maintained or institutionalized within a service setting’s ongoing, stable operations [10, 15]	Maintenance of intervention effectiveness over time. These data will be captured from clinical charts over a 24-h period. Duration (in months) that NICU continues to use the ImPaC Resource with fidelity. These data will be captured from the backend of the website	T3 (for INT group), and T4 (for both groups)
Other data	Description	Measure	Time points for data collection
Context evaluation by [1] staff nurses and [2] change team members	Factors describing the local organizational context	Alberta Context Tool (ACT) [14]. This is a reliable and valid tool that determines which elements of context facilitate and/or hinder successful KT outcomes [16]. It additionally captures a brief section on demographic data in addition to the 56 items and 5-point Likert scale answers for each item)	T1 (for staff nurses of both groups) and at commencement of the Resource (for both groups)

*CFIR* Consolidated Framework for Implementation Research, *INT* intervention, *KT* knowledge translation, *NICU* Neonatal Intensive Care Unit, *SP* standard practice

### Implementation (secondary) outcomes

Implementation outcomes (i.e. feasibility, fidelity, implementation cost, and reach) will be assessed to determine implementation effectiveness within groups. For the INT group following the completion of the intervention (at T2): (1) metrics will be captured by the Resource (e.g. user visits to the Resource, steps and relevant tasks completed, time interacting with each Step of the Resource and related activities, and expenses associated with these activities); and [2] focus group interviews will be conducted with members of the Change Team. For the SP group who elect to complete the six-month implementation of the Resource, implementation outcomes will be assessed using the same metrics captured by the Resource, as described above. No focus group interview will be undertaken for the SP group.

Sustainability will be assessed at T3 and T4 for INT group, and at T4 for SP group (See Table 1) through data retrieved from medical charts and metrics captured by the Resource.

### Organizational context (other) outcomes

Organizational context will be assessed with the Alberta Context Tool [28] to be completed by Staff Nurses at baseline (T1) and Change Teams (at commencement of the ImPaC Resource intervention) for both groups.

### Intervention (INT) group

NICUs randomized to the INT group will receive standard training and access to the Resource and will be invited to use it in their NICU over a six-month period. The Resource includes seven steps that are divided into a Plan Stage (Steps 1–4) and a Change Stage (Steps 5–7). The Change Stage incorporates a Plan-Do-Study-Act (PDSA) cycle that can be repeated multiple times within the six-month period until the desired practice change is achieved. In each step, the Change Team will:

- **Step 1:** Complete the “Change Team Checklist” to ensure members know of expected responsibilities that involve identifying a pain practice change and aim, planning and implementing interventions, and monitoring the change process and the unit’s progress.
- **Step 2:** Complete and reflect on the unit’s readiness for change using the “Readiness for Change Survey.” This survey is adapted from the ACT [28] and includes 34 items divided into five themes: communication; space; culture; feedback process; and leadership. Upon completion, the change team will receive feedback on their scores and guidance on strategies that can be used to improve any sub-optimal context areas.
- **Step 3:** Conduct an audit on 10 medical records for infants who have been in the NICU for > 24 h; this will provide unit baseline data on pain assessment and management practices. The Change Team will select the charts for a convenience sampling using a standardized approach (e.g. alphabetically at the beginning of each day, removing duplicates). The Change Team will identify a pain assessment or pain management practice as target for practice change based on the audit results.
- **Step 4:** Review of the evidence briefs about pain assessment or pain management included in the Resource. The Change Team will then develop an aim statement to precisely articulate the expected percentage of change to be achieved (e.g. 20%), and the interval of time required for achieving that change (e.g. over two months).
- **Step 5:** Select appropriate implementation strategies that will support the target evidence-based pain management or assessment practice change during PDSA cycles. Implementation strategies selected will be recorded on an activity planner. Educational and reminder implementation materials are downloadable and printable from the Resource. The Change Team will select tools and plan their use within the unit (e.g. target audience, intended number of individuals to reach, estimate cost and time for implementation).
- **Step 6:** Re-audit 10 infant medical records in as per Step 3. The results of this post-intervention audit will inform the percentage of change for the target practice change at the completion of each PDSA cycle.
- **Step 7:** Examine the effectiveness of implementation strategies and identify a new practice change target and associated implementation strategies for the next PDSA change cycle.

The Resource will be implemented locally by the NICU Change Team. The Plan Stage (Steps 1–4) is expected to be completed in one month. Each PDSA cycle in the Change Stage (Steps 5–7) is expected to be completed in approximately two months. Change Teams will be encouraged to complete two PDSA cycles of change over the six-month intervention period and to sustain use of the Resource for as long as they would like after the six-month intervention. Sustainability data will be collected at six and 12 months after intervention completion (Table 1).

### Standard Practice (SP) group

The SP group will continue as usual with their unit or institutional pain practices. Any implementation strategies implemented organizationally (e.g. new staff orientation) will be noted. The SP group will be offered the Resource following T2 outcome evaluations (e.g. six months after randomization) and be invited to utilize the Resource in a similar manner and time as the INT group. Sustainability data will be collected at six months after intervention completion (Table 1).

### Sample size

Data will be collected from 30 medical records of infants in each NICU. Assuming an inter-class correlation coefficient of 0.2, the variance inflation factor due to the cluster design is 6.8. With 16 NICUs, there will be an 80% power to achieve statistical significance at the 5% level, two-sided if the treatment arms differ by 0.67 standard deviations (SD; between-patient, within cluster), representing a moderate effect-size. Using estimates from prior studies [7] this would yield detectable differences of 0.33 for the binary outcome *pain assessed with a valid instrument*, and 0.27 for the binary outcome *any pain management*. A dropout rate of 10% of the sites is anticipated, therefore 18 units will be enrolled.

### Recruitment and assignment

Eligible level 2 and level 3 NICUs in Canada will be invited to participate via email from the lead research team to the Unit Manager and Medical Director, detailing a brief introduction to the study and recruitment video that will inform on study objectives, design, and timelines. Sites that express interest in participating will be offered a site visit to learn further details of the study. Once participation is confirmed, the recruited sites will receive assistance in applying for local institutional research ethics board (REB) approval. Baseline data collection on infant medical records will commence once institutional REB approval is granted and financial subcontracts are completed.

Participating NICUs will then be randomized to groups using a computer-generated random allocation sequence (randomize.net). Randomization will be stratified by NICU level (level 2 or level 3). Once units are randomized to the INT group, eligible HCP staff will be recruited to participate as a Change Team member and all eligible unit staff will be asked to complete the ACT. The Change Team for the SP group will be recruited and offered the ImPaC intervention after the six-month waiting period after unit randomization.

Data analysts will be blinded to group allocation. Change Team and NICU staff will not be blinded due to the visible nature of the intervention.

### Data collection procedures and methods

Baseline clinical data will be collected from 30 medical records of hospitalized infants by a trained research nurse or research assistant at each site before randomization at all NICUs (T1). If there are > 30 infants in the NICU, infant medical records will be randomly selected using a computer-generated random sequence. The ACT survey will be electronically distributed to all NICU nursing staff within each unit at baseline (T1). Two reminders will be sent out using the same methods at two-week intervals.

#### Intervention (INT) group

Three to five eligible HCPs within each NICU that is randomized to the INT group will be invited to participate on that NICU’s Change Team. Once identified and consented, each NICU group will receive a 1-h in-depth standardized training session on the Resource. At this session, Change Team members will be asked to complete the ACT survey and will be provided an individual login to access the Resource. The session will be delivered by a member of the lead site research team either in person (preferable) or by distance using a video conference program (e.g. Zoom).

During the six-month INT, the Change Team will independently navigate through the seven steps of the Resource with no implementation support from the lead research team. The lead site research staff will be available for questions should they arise but will not contact the site or provide implementation coaching; this distancing is important with respect to establishing external validity and sustainability of the Resource in the long term.

At the completion of the six-month INT, clinical data for 30 infant medical records in the NICU will be collected at each data collection point (T2–T4) by a trained research nurse or research assistant who is not involved in the implementation of the Resource. Implementation outcome data will be collected from metrics captured within the Resource and through semi-structured focus groups with Change Team members (one focus group per site), as described in Table 1. Focus groups will be conducted at each site in person (preferable) or via video conference by a trained facilitator from the lead site using an interview guide (Table 1). The Change Team will be encouraged to continue to use the Resource after the six-month INT.

#### Standard Practice (SP) group

Following randomization, units in the SP group will be advised to continue with their usual pain practices for six months.

Following data collection at T2, the SP units will be invited to use the Resource. They will follow the same procedures described for the INT group in terms of recruiting and training the Change Team members. The Resource will be monitored and evaluated in the same way as for the INT group. Data on clinical and implementation outcomes will be collected after a six-month period (T3); however, focus groups will not be conducted for units in the SP group.

Six months after completion of the Resource intervention (T4), data from 30 infant medical records per NICU will be collected. Resource metrics will also be examined.

Quantitative data (clinical, demographic, and organizational context data) will be collected and managed using Research Electronic Data Capture (REDCap™). Steps to promote data quality will be undertaken, including regular database review and range checks on data values. Implementation outcome data captured from embedded Resource metrics (e.g. feasibility, fidelity, implementation costs, and sustainability) will be captured and stored by the Resource website.

### Contamination and co-intervention

To avoid intervention contamination, we will use a cluster RCT with the hospital NICU as the unit of randomization. Where two or more eligible NICUs are co-located in one hospital or within one hospital organizational structure where there is significant overlap of staff and management, both sites will be randomized to receive the same intervention and will be counted as one NICU site for the purposes of the study analyses.

Co-intervention may occur if either group receives additional implementation strategies to improve practice and clinical pain outcomes (e.g. a hospital-based strategy to reduce procedural pain in infants) through initiatives outside the study. We will not interfere with any hospital-initiated activities that promote improvement in pain assessment or management within the NICU or the hospital during the trial. This information will be documented in focus groups following completion of the six-month INT. We will ask the SP groups to document this activity.

### Data management, analyses, and monitoring

#### Quantitative data

All primary analyses to determine intervention effectiveness will be conducted as intention to treat. The characteristics of infants admitted to participating NICUs and enrolled in the study will be summarized using descriptive statistics such as means and SD for continuous variables and medians and ranges for categorical variables. Given the lack of independence in outcomes due to sampling of multiple patients from the same NICU, inferential statistical methods that account for this clustering will be used. To assess the impact of the Resource on the clinical outcomes (proportion of infants with any validated pain assessment used and any pain management strategy used), the odds of a particular outcome will be compared between INT and SP groups as the odds ratio with 95% confidence interval and *p* value. Parameter estimation will be facilitated by a logistic regression model, with the clustering by NICU accomodated by estimation using Generalized Estimating Equations. The impact of contextual factors on clinical outcomes will be explored. Generalized estimating equation models for binary outcomes (i.e. logit link) will model implementation outcomes in both groups while including contextial covariates. All models will be assessed for goodness-of-fit. Imputation for missing data will be employed only if there is > 10% of missing data. Comprehensive training and database monitoring will be performed to minimize potential loss of data; in a previous study in which similar data was collected [16], missing data were minimal (< 5%). All analyses will be conducted using SAS v9.4 (Cary, NC, USA).

Analysis of implementation costs will be captured within Resource metrics which include quantifying resources required for its implementation over a six-month period—including: human resources (time spent on orientation and training, navigation through the Resource, meetings among Change Team members, implementation of the Resource either individually or in groups); equipment (e.g. laptop); materials (e.g. printing materials); and other related expenses—and will be captured by the Resource (Steps 5 and 7). The uncertainty of the findings will be explored. This analysis can provide evidence to inform scalability and sustainability of the Resource.

For organizational context data, descriptive statistics (e.g. means, SD) for each of the 10 ACT concepts will be reported. Outcome means for each NICU will be compared to the overall sample. We will also categorize the NICUs in a binary manner as high or low using quartiles. Analysis of variance and multiple comparison tests will be used to investigate differences in workplace environment characteristics relative to primary outcomes. If an adequate sample size is not achieved, then non-parametric methods (e.g. Kruskal–Wallis test) will be used. Regression modeling will be used to assess the influence of organizational factors on clinical pain practices to determine if units with more positive organizational context scores are more successful in adopting the resource and have more positive pain management practices.

#### Qualitative data

Focus group interviews will be conducted with the Change Team members from each NICU in the INT group and recorded using two digital recorders to minimize the potential for technical errors. Audio-recordings will be transcribed verbatim with all participants de-identified by an independent, professional transcriber. The interviewer and the qualitative data analyst will check the de-identified transcripts for accuracy by comparing the transcribed text to the audio recordings and correcting any errors or replacing missing data if possible. The CFIR constructs and domains of the ACT will be used to guide coding. A directed (deductive) seven-step approach to qualitative content analysis will be used [29, 30]. This approach permits determining a priori themes based on the conceptual framework of the study. To maintain rigor and establish trustworthiness of the data, we will analyze the focus group of each NICU separately and triangulate data from all data collection strategies, undertake reflection to monitor impressions as they emerge, and create an audit trail to keep track of decisions made during the analyses. Categories, patterns, and themes will be extracted from the transcribed focus groups and compared across sites.

### Monitoring

No interim analyses or audits of clinical outcomes data are planned. Given that “inactivity or missing data” in the Resource website by the Change Team will be analyzed as part of the implementation outcomes (e.g. feasibility, fidelity, reach, sustainability), and given there are no anticipated potential adverse events to participating in this project, data and safety monitoring by a separate committee will not be required.

There are no anticipated potential adverse events to HCPs participating in this project. Users are responsible for engaging with the Resource material and activities within their clinical settings and any clinical outcomes or interventions that may arise from this activity. Users are advised to follow institutional policies and guidelines to implement any pain practice change.

Any request from a participating unit or individual change team member to discontinue their participation in the trial would be honored and the reason(s), if provided, noted. Data collected up to the point of discontinuation would be included in the analyses.

### Dissemination

We plan to share the results of the trial with researchers, HCPs, decision-makers, and parent groups and families. Findings will be disseminated first to those individuals in the participating sites and then more broadly via social media, presentations, and plain-text clinical guidelines, tip-sheets, and other messaging tailored for the target audience. Peer-reviewed publications and presentations at national/international conferences will target academic audiences.

## Discussion

To our knowledge, this is the first web-based, multifaceted implementation Resource for self-administration by a small group of change agents to foster change and improvement of pain practices in infants. Since EPIQ was described as useful but time-intensive for users [15, 16] in earlier studies, the goal of the Resource is to provide a feasible, acceptable, sustainable, and economically attractive resource to facilitate practice change. Exploring clinical and implementation effectiveness for the Resource will inform researchers, decision-makers, and clinicians about this approach to practice change towards better assessment and management of neonatal pain as well as health outcomes.

### Strengths and limitations

A significant strength of this research is the use of an effectiveness-implementation hybrid type 1 design. This design evaluates clinical effectiveness while gathering information on implementation outcomes and has the potential to speed and improve the translation of evidence-based pain practices into clinical care [31]. Clinical outcomes have been clearly defined based on prior studies [15, 16]. Efforts were made to select the most relevant implementation outcomes and to use the best definitions available [19, 32]. Quantitative and qualitative data will be triangulated to generate a comprehensive understanding of implementation outcomes relative to the clinical outcomes.

Clinical documentation (charting) of pain practices may not always accurately reflect the pain practices in use within the participating hospital units. Thus, incomplete documentation may be a limitation for clinical outcome data collection. In terms of pain assessment, as there is no gold standard measure for the neonatal and infant population, we expect some variation in the validated measures implemented at each site. For the primary outcome, data will be analyzed as dichotomous (yes/no) for whether any validated measure was implemented. More specific information on pain measure names and pain intensity scores documented will be collected and converted to a standardized score for secondary comparison purposes.

Sustaining the impact of practice change is an important consideration in intervention and implementation research [33], but no clear definition of sustainability has been established to date. For this study, sustainability encompasses five constructs recently described in the literature, including maintenance of: (1) the intervention after a defined period; (2) implementation strategies; (3) individual behavior change; and that (4) the program and behavioral change may evolve or adapt while (5) continuing to produce benefits [34].

Organizational context is an important factor influencing implementation of practice change and this may vary considerably across the units included. Given previous experience, the anticipated response rate from nursing staff in each unit for completing the ACT survey is in the range of 30%–50%. The influence of organizational context on clinical and implementation outcomes will be carefully analyzed.

### Trial status

This is protocol version 2, 6 December 2018. The first neonatal unit was recruited in April 2019 and recruitment has been completed in the fall of 2019. Study activation procedures have started in the fall of 2019.

## Supplementary information

**Additional file 1.** Standard Protocol Items: Recommendations for Interventional Trials – Child Health Extension Checklist (SPIRIT-C 2019)

## Data Availability

Not applicable.

## Abbreviations

ACT: Alberta Context Tool
CFIR: Consolidated Framework for Implementation Research
EPIQ: Evidence-based Practice for Improving Quality
HCP: Healthcare professional
ImPaC: Implementation of Infant Pain Practice Change
INT: Intervention
NICU: Neonatal Intensive Care Unit
PDSA: Plan-Do-Study-Act Cycle
REDCap: Research Electronic Data Capture
SAS: Statistical Analysis System
SP: Standard practice
